# Phenotypic presentation of *MEN1* c.758delC (p.Ser253Cysfs^*^28) pathogenic variant: a case report

**DOI:** 10.1093/omcr/omae111

**Published:** 2024-09-22

**Authors:** Antonio Mancini, Paola Concolino, Edoardo Vergani, Alessandro Oliva, Giuseppe Macis, Emanuela Traini, Esther Diana Rossi

**Affiliations:** Operative Unit of Internal Medicine, Endocrinology and Diabetology, Department of Translational medicine and surgery, Fondazione Policlinico Universitario “Agostino Gemelli”, Scientific Institute for Research, Hospitalization and Healthcare (IRCCS), Largo Agostino Gemelli, 8, 00168, Rome, Italy; Clinical Chemistry, Biochemistry and Molecular Biology Operations (UOC), Fondazione Policlinico Universitario ‘Agostino Gemelli’, Scientific Institute for Research, Hospitalization and Healthcare (IRCCS), Largo Agostino Gemelli, 8, 00168, Rome, Italy; Operative Unit of Internal Medicine, Endocrinology and Diabetology, Department of Translational medicine and surgery, Fondazione Policlinico Universitario “Agostino Gemelli”, Scientific Institute for Research, Hospitalization and Healthcare (IRCCS), Largo Agostino Gemelli, 8, 00168, Rome, Italy; Operative Unit of Internal Medicine, Endocrinology and Diabetology, Department of Translational medicine and surgery, Fondazione Policlinico Universitario “Agostino Gemelli”, Scientific Institute for Research, Hospitalization and Healthcare (IRCCS), Largo Agostino Gemelli, 8, 00168, Rome, Italy; Department of Diagnostic Imaging, Oncological Radiation Therapy and Hematology Fondazione Policlinico Universitario ‘Agostino Gemelli’, Scientific Institute for Research, Hospitalization and Healthcare (IRCCS), Largo Agostino Gemelli, 8, 00168, Rome, Italy; Endocrine Surgery Unity, Ospedale San Carlo di Nancy GVM, Via Aurelia, 275, 00165, Rome, Italy; Division of Anatomic Pathology and Histology, Catholic University of Sacred Heart, Largo Agostino Gemelli, 8, 00168, Rome, Italy

**Keywords:** MEN1, novel mutations, genetics, hyperparathyroidism, pancreatic NET, pituitary adenomas

## Abstract

MEN1 is a rare syndrome caused by mutations in the MEN1 gene. We describe a clinical case of MEN1 syndrome associated with a recently discovered pathogenic mutation of MEN1 gene. A 32-year-old man with a history of osteopenia, nephrolithiasis, hypercalcemia and hypophosphatemia, impaired fasting glucose, and asthenia was admitted to our outpatient unit. Primary hyperparathyroidism, sustained by three hyperplastic parathyroid glands, was diagnosed. Prolactin- and GH-secreting adenomas were ruled out. After undergoing subtotal parathyroidectomy, the patient was diagnosed with non-functioning pituitary adenoma, three pancreatic lesions, and Cushing syndrome sustained by left adrenal adenoma. The patient underwent left adrenal surgery; somatostatin analogue lanreotide was started for the pancreatic lesions; the pituitary adenoma, being small and non-secreting, was not treated. A genetic test was performed to confirm the diagnosis of MEN1 syndrome, finding an association with a recently discovered mutation: the (NM_130799.2):c.758delC (p.Ser253Cysfs^*^28) in exon 4.

## Introduction

Multiple Endocrine Neoplasia Type 1 (MEN1) is a rare autosomal dominantly inherited endocrine syndrome [1], which determines the development of several endocrine tumors, with a high incidence of primary hyperparathyroidism (pHPT), pituitary adenomas (PA) and neuroendocrine tumors (NET) of the pancreas (PanNET) and duodenum (dNET) [2].

The *MEN1* gene consists of 10 exons and is located on chromosome 11q13. It codes for the protein menin, a nuclear scaffold protein that organizes chromatin remodeling, thus regulating gene transcription and acting as an oncosuppressor; its loss of function mutations determine menin alterations and, as a consequence, the MEN1 syndrome [3].

Reported, more than 700 pathogenic and likely pathogenic variants have been reported on the gene: the frameshift mutations represent the highest rate (42%), while missense variants show a frequency of 25.5%, nonsense mutations 14%, splice-site mutations 10.5%, in-frame del/ins 5.5% and gross deletions the remaining 2.5%. In addition, more than 1000 variants with uncertain significant (VUS) have been reported [4–7].

The variants are located at multiple loci of the *MEN1* gene without obvious hotspots, although some similar mutations have been described in seemingly unrelated families, as happens with the founder effect [8].

Usually, most variants are detected by sequence analysis, but since 1%–2% of these are deletions [6], copy number variation (CNV) analysis should also be considered in diagnostic DNA testing.

Here, we describe for the first time a clinical case of a patient with a recently discovered pathogenic variant of *MEN1* associated with four different endocrine neoplasms.

## Case report

A 32-year-old Italian male patient was admitted to our outpatient service for osteopenia and a history of nephrolithiasis in hypercalcemia and hypophosphatemia. The patient also had a history of hypertension, impaired fasting glucose and asthenia. His father was affected by familiar hypercalcaemic hypocalciuria (FHH). Table 1, Section 1, shows the results of the admission laboratory evaluation (Table 1). Primary hyperparathyroidism (pHPT) was confirmed both biochemically and morphologically. ^99^Technetium-sestamibi (99Tc-MIBI) scintigraphy showed an increase in the uptake of 99Tc-MIBI near the left thyroid lobe. The diagnosis was ruled out by evaluating catecholamines, metanephrines, normetanephrines, vanilmandelic acid, and dopamine in 24-hour urine collection. Insulin like growth factor (IGF) -1 and prolactin (PRL) were evaluated as rule out exams for the most common pituitary functioning adenomas and both resulted normal (Table 1, Section 1).

**Table 1:** Laboratory results (before parathyroidectomy, before and after unilateral adrenalectomy)

	**1. Before Parathyroidectomy**		**2. After Parathyroidectomy—before Adrenalectomy**		**3. After Unilateral (left) Adrenalectomy**	**Normal Range**
Calcium (mg/dl)	12.8		8.9			8.6–10.2
Albumin (g/l)	41					34–48
Phosphate (mg/dl)	1.8					2.5–4.5
Vitamin D (ng/ml)	26.7		25		43.7	31–100
PTH (pg/ml)	274.7		26		39.6	14–72
Urinary 24 h Calcium (mg/l)	120	117				50–300
ACTH (pg/ml)			24		55	10–55
Cortisol (ng/ml)			160		137	60–220
Sodium (mmol/l)			143		141	135–145
Potassium (mmol/l)			4.1		4.5	3.5–5.5
PRL (ng/ml)	18.7		21.5			3.5–15.5
IGF-1 (ng/ml)	213		195			80–330
Chromogranin A (ng/ml)			207		136.5	19.4–98.1
Salivary Cortisol (ng/ml)			0.05	0.03		<0.11
Serum Cortisol after DEX1 (ng/ml)			76	19		<18
Urinary 24 h Cortisol (μg/24 h)			72	43		<70

PTH: parathormone, ACTH: adrenocorticotropic hormone, PRL: prolactin, IGF-1: insulin-like growth factor-1, DEX1: overnight dexamethasone 1 mg test.

The patient underwent subtotal parathyroidectomy (left superior and inferior, right superior) since three hyperplastic parathyroids were found during the surgery. The intraoperative dose of PTH showed a significant decrease from 1285 pg/ml to 107 pg/ml. Table 1, Section 2, shows good control of serum calcium after surgery (Table 1). The tissue histology showed parathyroid hyperplasia without elements of atypia.

The persistence of asthenia and slightly elevated blood pressure and glucose levels prompted us to re-evaluate pituitary hormones. No signs of pituitary hyper/hyposecretion were detected, except for a single finding of high ACTH level and altered circadian rhythm. Table 1, Section 2, shows the Cushing syndrome screening test, which appeared to support a possible Cushing disease (positive DEX1 plus slightly positive 24-hour urinary cortisol plus ACTH levels >5 pg/ml).

**Figure 1:**
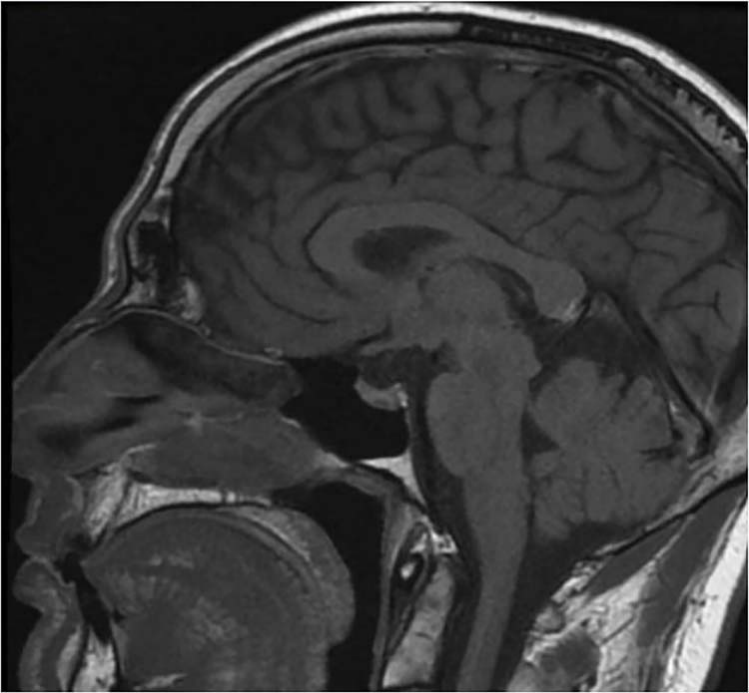
MRI in T1. The detailed MRI study of the diencephalon-pituitary region shows a thinned pituitary gland lying on the sellar floor, with a concave profile. A small expansive, oval, formation with a maximum diameter of 8 mm and height of about 3 mm is located in the median/left paramedian and basal portion of the gland.

**Figure 2:**
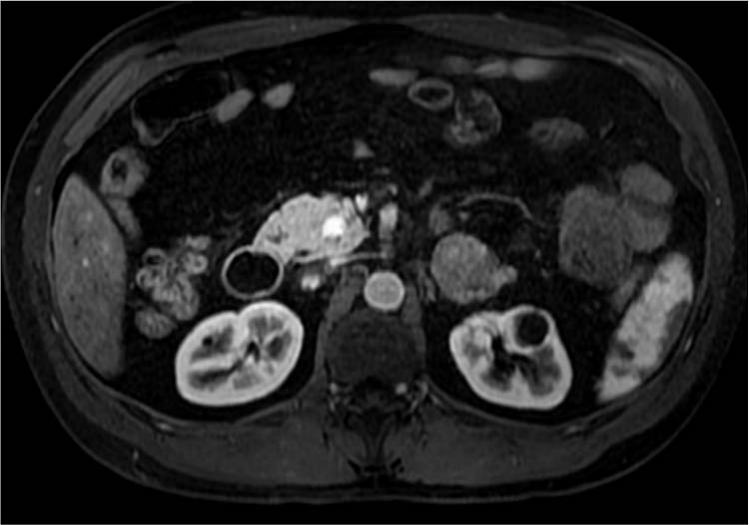
CT scan in the arterial phase after contrast injection. The highly hyperintense 13 mm cephalopancreatic hypervascular nodule characteristic of neuroendocrine tumors is clearly noticeable. In the lateral lip of the left adrenal gland, a 39 mm nodule with clear margins showing strong T1 signal reduction out of phase and inhomogeneous, weak, enhancement after contrast medium (typical of adrenal adenomas) can be seen.

Pituitary magnetic resonance imaging (MRI) showed an 8 mm lesion suggestive of microadenoma (Fig. 1). However, the second biochemical assessment of Cushing syndrome was equivocal and conflicting. The concomitant detection of a high level of chromogranin A led us to perform an abdominal CT under suspicion of NET. CT showed three pancreatic lesions, all under 2 cm; additionally, it showed a 4 cm lesion of the medial lip of the left adrenal gland (Fig. 2). ^68^Ga PET-CT was performed, showing high uptake of the radionuclide, suggesting a high expression of somatostatin receptors, in the pancreatic area and the left adrenal gland. Ultrasound endoscopy and subsequent biopsy of pancreatic lesions confirmed the diagnosis of panNET G1. Regarding the left adrenal nodule, I-norcholesterol scintigraphy revealed unilateral uptake in the left adrenal gland.

**Figure 3:**
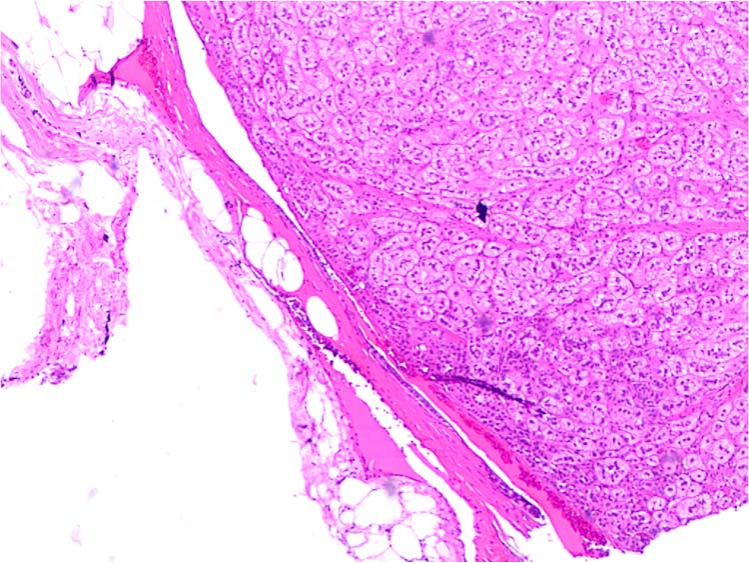
The picture shows details of an adrenal adenoma characterized by cellular elements with small size, few atypical nuclei and clear cytoplasm. H&E 400X.

**Figure 4:**
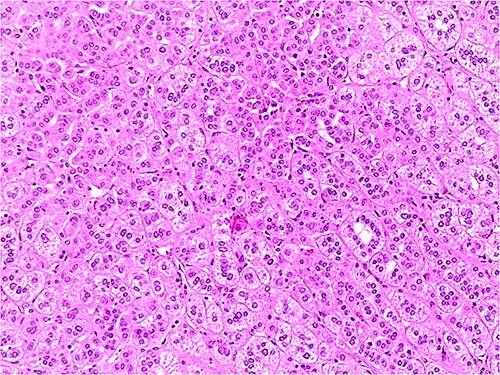
The picture shows details of the same adrenal adenoma with the peripheral capsular border. No evidence of invasion. H&E 200X.

The patient underwent left adrenal surgery due to the dimension of the nodule. Histology confirmed left adrenal adenoma (Figs 3 and 4). The patient had an uneventful postoperative course. After adrenalectomy, improved blood pressure and serum glucose were recorded. As expected, ACTH levels were kept at the upper level of the normal range. (Table 1, Section 3). No substitutive treatment was needed.

The somatostatin analogue lanreotide (30 mg every 28 days) was started and periodic abdominal CT and hormonal follow-up are performed.

The concomitant presence of pHPT, multifocal panNETs, nonfunctioning pituitary microadenoma and left adrenal adenoma is clearly suspicious for MEN. Genetic testing identified the *MEN1* pathogenic variant (NM_130799.2):c.758delC (p.Ser253Cysfs^*^28) in exon 4.

The patient is now in six-month follow-up. The three pancreatic lesions are currently stable under lanreotide. Pituitary adenoma did not increase during the years of follow-up. Recently, an increase in serum and urinary 24 h calcium has been detected. PTH is slightly increased while kidney function and vitamin D are in the normal range. It is therefore plausible that the remaining parathyroid is hyperplastic as the three that have been removed. Given the slight increase in calcium and PTH, the patient was told to increase daily hydration. In case of worsening of serum calcium values, calcimimetics will be considered. The father and the mother of the patient refuse to perform genetic testing. The patient is now looking for a pregnancy with his wife. She was tested negative for menin mutations.

## Discussion

The c.758delC variant, as reported in our patient, creates a premature translational stop signal (p.Ser253Cysfs^*^28) in the *MEN1* gene. It is expected to result in an absent or disrupted protein product. In fact, it shortens the menin by only 278 amino acids, lacking all three nuclear localization signals (NLSs). Loss-of-function variants in *MEN1* are known to be pathogenic. The variant is not present in population databases (gnomAD). This premature translational stop signal has been observed for the first time in individuals with MEN1-related disease (called g.868delC) as reported in the Italian multicentre MEN1 patients database [9]. In this paper, it is noted that a direct genotype–phenotype correlation is not apparent.

In general, frameshift and nonsense mutations of the *MEN1* gene are associated with aggressive gastro-enteropancreatic NETs (GEP NETs) and specially panNET [2]. There is no agreement on this point. Marini reported that patients with GEP-NETs had a significantly higher frequency of nonsense rather than frameshift or missense mutations [9]. The DutchMEN study group reported that patients with clinical MEN1 who lack a specific gene mutation usually have a later insurgence of disease, often developing two lesions, with a life expectancy similar to that of the general population; their family history is usually null when researching for previous cases of MEN; finally, they frequently show uniglandular pHPT [10, 11].

The novelty of the present report relies on the association between the *MEN1* pathogenic variant (NM_130799.2):c.758delC (p.Ser253Cysfs^*^28) in exon 4 and a clinical phenotype characterized by pHPT, non-functioning pituitary microadenoma, left adrenal adenoma and multifocal pancreatic NETs. To our knowledge, this is the first report of this association in the literature.

## Consent

Written informed consent was obtained.

## Guarantor

Antonio Mancini.
